# Rapid and repeatable shifts in life‐history timing of *Rhagoletis pomonella* (Diptera: Tephritidae) following colonization of novel host plants in the Pacific Northwestern United States

**DOI:** 10.1002/ece3.1826

**Published:** 2015-11-26

**Authors:** Monte Mattsson, Glen R. Hood, Jeffrey L. Feder, Luis A. Ruedas

**Affiliations:** ^1^ Department of BiologyPortland State University1719 SW 10^th^ Ave.Science Building OnePortlandOregon97201; ^2^ Department of Biological SciencesUniversity of Notre Dame100 Galvin Life SciencesNotre DameIndiana46556

**Keywords:** Allochronic isolation, apple maggot fly, biodiversity, ecological speciation, hawthorn, host race formation, phytophagous insects

## Abstract

Host shifts of phytophagous insect specialists to novel plants can result in divergent ecological adaptation, generating reproductive isolation and potentially new species. *Rhagoletis pomonella* fruit flies in eastern North America underwent a host shift ~160 ya from native downy hawthorn (*Crataegus mollis*) to introduced, domesticated apple (*Malus domestica*). Divergent selection on diapause phenology related to the earlier fruiting time of apples versus downy hawthorns resulted in partial allochronic reproductive isolation between the fly races. Here, we test for how rapid and repeatable shifts in life‐history timing are driving ecological divergence of *R. pomonella* in the Pacific Northwestern USA. The fly was introduced into the region via larval‐infested apples 40–65 ya and now attacks native black hawthorn (*Crataegus douglasii*) and introduced ornamental hawthorn (*Crataegus monogyna*), in addition to early‐ and late‐maturing apple varieties in the region. To investigate the life‐history timing hypothesis, we used a field‐based experiment to characterize the host‐associated eclosion and flight activity patterns of adults, and the feeding times of larvae at a field site in Vancouver, Washington. We also assessed the degree to which differences in host‐fruiting time generate allochronic isolation among apple‐, black hawthorn‐, and ornamental hawthorn‐associated fly populations. We conclude that host‐associated fly populations are temporally offset 24.4% to 92.6% in their seasonal distributions. Our results imply that *R. pomonella* possesses the capacity for rapid and repeatable shifts in diapause life history to match host‐fruiting phenology, which can generate ecologically based reproductive isolation, and potentially biodiversity in the process.

## Introduction

Ecological speciation is initiated when divergent adaptation to novel habitats generates reproductive isolation (Schluter 2001; Rundle and Nosil 2005). Phytophagous insect specialists may be particularly prone to speciate ecologically when they shift and differentially adapt to new host plants (Bush 1969; Harrison 1985; How et al. 1993; Abrahamson et al. 1994; Horner et al. 1999; Via 1999; Wood et al. 1999; Groman and Pellmyr 2000; Via et al. 2000; Berlocher and Feder 2002; Drès and Mallet 2002; Funk et al. 2002). When divergent selection pressures between alternate host plants are strong, migrants between habitats and hybrids of mixed ancestry will suffer reduced fitness, generating ecologically based reproductive isolation that can potentially initiate speciation even in the face of gene flow (Bush 1969; Feder et al. 1988; Berlocher and Feder 2002; Drès and Mallet 2002; Funk et al. 2002; Nosil 2012; Sim et al. 2012).

Apple‐ and downy hawthorn‐infesting populations of the fruit fly *Rhagoletis pomonella* (Walsh) (Diptera: Tephritidae) have been hypothesized to represent an example of incipient ecological speciation with gene flow (Berlocher and Feder 2002). Sometime in the mid‐1850s, *R. pomonella* shifted in the eastern United States (US) from its native host, downy hawthorn, *Crataegus mollis* ([Torr. & A. Gray] Scheele), to form a new “host race” on domesticated apple trees (*Malus domestica* [Borkh.]) after apples were introduced from Europe ~400 ya (Walsh 1867; Bush 1966; Feder et al. 1988; McPheron et al. 1988). Host races are hypothesized to be the initial stage of ecological speciation, representing partially reproductively isolated populations that owe their isolation to divergent host‐ or habitat‐associated adaptations (Diehl and Bush 1984; Drès and Mallet 2002).

**Figure 1:**
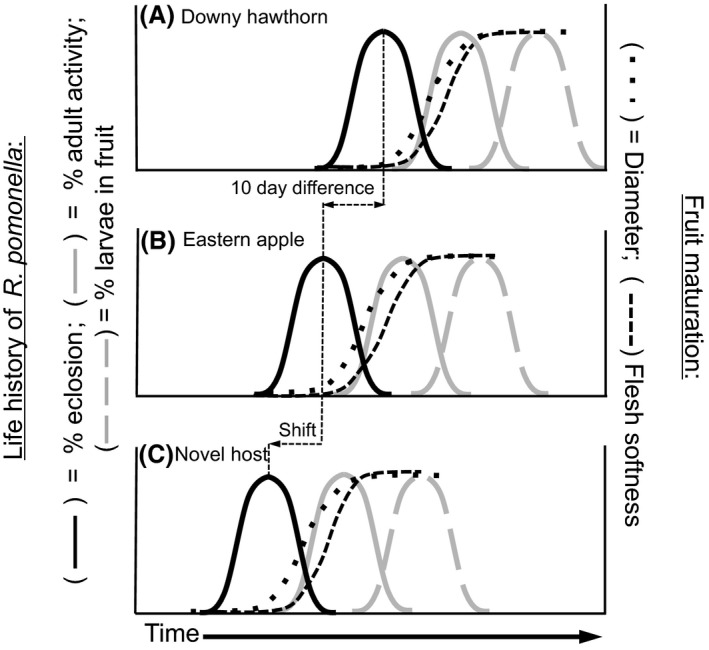
Allochronic premating isolation resulting from differences in eclosion times (idealized here for clarity) of (A) downy hawthorn‐ and (B) apple‐associated populations of *Rhagoletis pomonella* in the eastern USA. Panel (C) illustrates a hypothetical shift resulting from novel colonization of a temporally adjacent, earlier fruiting host. Eclosion of adults strongly correlates with natal fruit ripening (quantified using fruit diameter and flesh softness), followed by mating and oviposition (“Adult Activity”) and larval development within fruit. Flies reach sexual maturity 7–10 days posteclosion, helping to explain why eclosion curves slightly precede fruit maturation. Modified from Bush (1969).

One key ecological adaptation reproductively isolating and genetically differentiating the *R. pomonella* host races is the relationship between host‐fruiting phenology and fly diapause (Fig. 1). Phytophagous insects must time their life cycle to coincide with environmental conditions when plant resources are plentiful for feeding, mating, and oviposition (Tauber et al. 1986; Denlinger 2002). Life‐history timing is particularly important for univoltine insects like *Rhagoletis* that are host specialists, where short‐lived adults reproduce once a year and then die, and their offspring feed and develop on only one or a few specific plant species. In this case, the seasonal window when hosts are present and in optimal condition can be limited. Mismatches between the timing of host and parasite life cycle events can therefore have disastrous fitness consequences for a univoltine specialist insect, as it will be active at a time when both its host is absent and climatic conditions are potentially unfavorable for its survival (Tauber et al. 1986; Denlinger 2002).

Fruit on apple varieties favored by *R. pomonella* ripen on average 3–4 weeks earlier than those on downy hawthorn (Feder et al. 1993, 1994) (Fig. 1). Because *R. pomonella* live for a maximum of perhaps 1 month in nature, apple and downy hawthorn flies must differentially time their life histories to match the difference in the fruiting phenologies of their respective host plants. They accomplish this through variation in the timing of the overwintering pupal diapause. Apple flies have been shown to differ genetically from downy hawthorn flies in the intensity to which they initiate and terminate (break) diapause. Most notably, apple flies eclose as adults 10 days earlier on average than downy hawthorn flies to coincide with the earlier fruiting time of apples (Feder et al. 1993, 1994, 1995; Feder 1995; Egan et al. 2015). Due to the limited life span of adults, the difference in eclosion time results in partial allochronic mating isolation between the apple and downy hawthorn host races (Feder et al. 1993, 1994). In addition, a degree of postzygotic isolation also likely exists due the diapause timing of hybrids being less well‐adapted to exploiting apple or hawthorn fruit resources as those of parental genotypes (Smith 1988).

**Figure 2:**
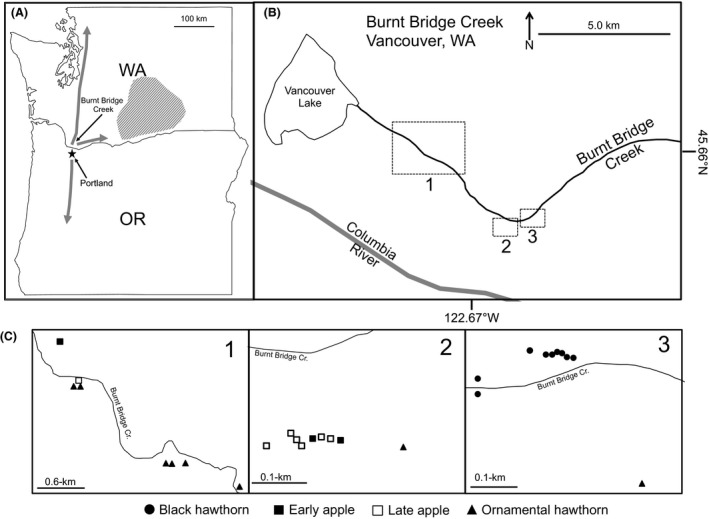
(A) *Rhagoletis pomonella* were first detected in Portland, Oregon, in 1979 (AliNiazee and Penrose 1981) and have spread north and south in Washington and Oregon, as well as into the Columbia River Gorge (gray arrows) toward Washington's fruit growing regions (shaded area). (B) The Burnt Bridge Creek Greenway, Vancouver, WA, including surrounding bodies of water. Sample sites were located in three general areas, indicated by boxes 1–3. Panel (C) details each of the three boxed areas of (B) to show the specific arrangement of eclosion tents. Inset photograph depicts one of the eclosion tents.

In this study, we examine the rapidity and repeatability of the evolution of diapause life‐history timing in a unique circumstance involving host‐associated populations of *R. pomonella* in the Pacific Northwest (PNW) region of the USA. *Rhagoletis pomonella* is native to the eastern USA. However, the fly was discovered infesting an apple tree in the yard of a homeowner in the Portland, Oregon (OR) area in 1979 (AliNiazee and Penrose 1981). In the 1980s, *R. pomonella* was found (had spread) north and south in Oregon and Washington (WA) on the western side of the Cascade Mountains and into the Columbia River Gorge and other passages in the Cascades encroaching on the commercial apple‐growing region of central WA (Dowell 1988; see arrows in Fig. 2A), where it is now a quarantine pest threatening the $2.25 billion annual apple industry of the state (Mertz et al. 2013). The rapid spread of the species is not surprising. Individual flies can disperse 0.2–4.5 km (Maxwell and Parsons 1968; Sharp 1978). Perhaps more importantly, the long‐distance transport of infested apples by humans was likely responsible for much of the spread. A single apple can harbor >10 larvae, which as adults are each capable of laying >200 eggs during a single lifetime. Exponential growth in this fashion will quickly lead to rapid dispersal of this species.

It is possible that *R. pomonella* was present earlier. For example, apple maggots were detected in a single “lot” of apples shipped from Portland, OR to California in 1947 (AliNiazee and Westcott 1986). However, the host tree from which the apples were harvested was identified and monitored for adult flies using yellow sticky traps, along with several adjacent properties in 1948 (the following year). Only two adult apple maggot flies were found, yet it remains unknown whether they were truly apple maggot flies, or perhaps instead adult snowberry maggot flies (*R. zephyria* Snow), which are morphologically cryptic with *R. pomonella* – the subtle differences in genitalia and wing shape were not known at this time. To further complicate matters, snowberry bushes (*Symphoricarpos albus* Blake) were found growing across the street from the purported host apple tree (AliNiazee and Westcott 1986). Another early specimen was taken from near Rowena, OR, in 1951 and was identified as being an apple maggot fly (Dowell 1988). However, *R. pomonella* remained undetected throughout the entire region until the late 1970s, despite extensive trapping studies designed to monitor those and other pests of agriculture in the area, such as *R. indifferens* (western cherry fruit fly) and *R. completa* (walnut fruit fly), so it is therefore unlikely that either of these early introductions were successful. Finally, genetic, behavioral, and distributional data support the hypothesis that the fly was recently introduced into the PNW via the human transport of larval‐infested apples (McPheron 1987; Dowell 1988; Linn et al. 2012; Sim et al. 2012; Hood et al. 2013; Sim 2013).

In addition to apple, *R. pomonella* also currently infests native black hawthorns, primarily *C. douglasii* (Lindl.), but also occasionally *C*. *suksdorfii* (Sarg.) Kruschke at low levels and introduced ornamental hawthorn (*Crataegus monogyna* [Jacq.]) (AliNiazee and Westcott 1987; Tracewski et al. 1987; Dowell 1988; Yee 2008; Yee et al. 2012; Hood et al. 2013; Sim 2013). Thus, the possibility exists that *R. pomonella* has shifted from apple within the last ≤65 years and is in the process of ecologically differentiating on novel black and ornamental hawthorn hosts in the PNW on an even more recent timescale than what occurred in the opposite direction in the eastern USA.

Interestingly, the fruiting phenologies of hawthorns in the PNW bracket that of apple. Fruit on ornamental hawthorns in the PNW, like those of downy hawthorn in the eastern USA, ripen later than sweeter apple varieties most favorable for *R. pomonella* larval development and survivorship. In contrast, native *C. douglasii* (black hawthorn hereafter) is an early‐fruiting host that precedes ornamental hawthorns and most of even the earliest fruiting apple varieties in the PNW. The native *C*. *suksdorfii*, another “black hawthorn” species in the PNW, is relatively rarely attacked by *R. pomonella* (Yee et al. 2012) and generally has a fruiting phenology comparable to that of *C. douglasii*, although it can vary earlier or later locally (J. L. Feder, pers. obs.). The existence of a variety of hawthorn‐associated forms of *R. pomonella* in the PNW, coupled with presence of the apple race in the eastern USA, therefore suggests that the fly has repeatedly shifted its diapause life history to attack novel host plants with different fruiting times. Moreover, the documented introduction of *R. pomonella* into the PNW within the last 40–65 years provides a historical context to infer that diapause life‐history shifts can occur rapidly. Finally, the different hawthorn‐associated populations of *R. pomonella* in the PNW imply that the fly can readily change the timing of its life history to attack novel plants that fruit not only earlier in the field season, as is the case for the downy hawthorn to apple shift in eastern USA, but also later, as well.

Here, we conduct a series of field‐based studies to test the hypothesis that variation in host plant fruiting phenology is a rapid and repeated driver of diapause life‐history adaptation generating reproductive isolation among host‐associated populations of *R. pomonella* in the PNW. To accomplish this, we first characterize the fruit ripening times of black hawthorn, apple, and ornamental hawthorn trees at field site in Vancouver, WA, where these alternative host plants co‐occur sympatrically. We then compare these seasonal windows of fruit resource availability with adult eclosion, flight activity, and oviposition times of the associated black hawthorn‐, apple‐, and ornamental hawthorn‐infesting *R. pomonella* populations at the site. Taken together, the results allow us to estimate the degree of temporal overlap and premating allochronic isolation generated by differences in diapause timing across multiple life‐history stages of the fly, potentially contributing to the formation of new hawthorn‐associated forms of *R. pomonella* in the PNW.

## Materials and Methods

### Life history

*Rhagoletis pomonella* are univoltine and adults live ~30 days (Dean and Chapman 1973; Boller and Prokopy 1976). After mating on or near host fruit, females oviposit just below the outer skin surface of ripe fruit on trees. After eggs hatch, larvae feed within host fruit for 2–3 weeks and undergo three larval instars. When fruits abscise from trees, larvae emerge from the fruit, burrow 1–5 cm into the ground, form puparia, and then enter a facultative pupal diapause in which they overwinter. The following summer, flies have terminated diapause and eclose as adults, completing their univoltine life cycle (Dean and Chapman 1973; Boller and Prokopy 1976; Bush 1994).

### Fruit ripeness

To determine the temporal windows and seasonal overlap of host resources for mating and oviposition, we characterized the fruit ripening phenologies of black hawthorn (*C. douglasii*), ornamental hawthorn, and early‐ and late‐maturing varieties of apples, the primary hosts of *R. pomonella* in the PNW, at a field site where the trees co‐occur in the Burnt Bridge Creek Greenway (45°37.9′N; 122°37.1′W) in Vancouver, Washington, USA, in the summer of 2013 (Fig. 2). We quantified fruit ripening by measuring the size and firmness of fruits from nine different black hawthorn trees, seven ornamental hawthorn trees, three early‐maturing apple trees, and seven late–maturing apple trees. Data from the early‐ and late‐maturing apple varieties were considered separately in all subsequent statistical analyses. A similar intraspecific bimodality was not observed for either black or ornamental hawthorn trees.

Previous research has indicated that fruit firmness is an important indicator of the susceptibility of host fruit to attack by *R. pomonella*. For example, Messina and Jones (1990) found that populations of *R. pomonella* in Utah do not begin infesting black hawthorn fruits until penetration resistance drops below 60 kg per cm^2^. Whether the physical resistance of the fruit prohibits oviposition, or reduced firmness (hereafter “softness”) simply coincides with rises in concentrations of sugars, tolerable pH levels, or other variables of host fruit acceptability, remains unknown. Regardless, excessively firm fruit present a biological obstacle to oviposition, as females must perforate the skin of fruit to lay eggs without damaging their ovipositors. In this regard, Dean and Chapman (1973) showed that softer apple fruits require approximately 10 sec for females to successfully penetrate fruit for oviposition, as opposed to 3 to 4 times longer for hard fruit. We therefore focused on fruit softness and size as metrics of fruit ripeness in the current study.

To gauge fruit ripeness, we did not begin collecting fruits until it was biologically relevant for a given host (i.e., until fruits were no longer too hard, green, and small to provide viable larval habitat). Black hawthorn fruits were collected from trees three times per week from 4 June to 17 July. Ornamental hawthorn fruits were sampled from one to two times per week from 3 July to 19 September. Five to seven fruits were collected from each hawthorn tree on each sample date (approximately 60 fruit total per hawthorn host species per sampling date; Table S1). Because apples were less numerous than hawthorn fruits, a slightly different sampling method was used to prevent the depletion of apples on source trees. For apples, five fruit were collected per tree every 7–10 days from 19 June to 19 September (Table S1). Equal numbers of fruits were collected randomly from each tree on each sampling date to reduce bias in the analysis and generate a sample of the spectrum of ripeness at the time of collection, as fruits on a single tree can ripen at unequal rates. To accomplish this, we repeatedly generated random numbers between 0 and 360 and treated a tree's canopy as a circle. The random number represented the degree from north (clockwise) at which the fruit first seen facing the trunk at eye‐level (1.7 m) was picked. Apples were sampled in a similar manner except that we also randomized the horizontal (distance from tree trunk) and vertical (distance from lowest to highest reachable canopy) location of harvesting by generating numbers from one to five and dividing the height and width of trees into 20% subsections. The maximum height sampled in an apple tree was 6.8 m.

The size and firmness of fruits were recorded within 2 h of sampling. For size, the transverse diameter of the widest portion of the fruit halfway between the calyx and peduncle was measured using a ruler to the nearest 0.5 mm (hawthorn) or 0.5 cm (apple). Oviposition and/or larval feeding are impeded by excessive firmness of fruits (Dean and Chapman 1973). Thus, a penetrometer (Firmness Tester FT‐02^®^ for hawthorn; FT‐327^®^ for apple, QA Supplies, LLC, Norfolk, VA) affixed with either an one mm probe (for hawthorns) or an 11 mm probe (for apples) was used to measure the resistance of a fruit's surface to penetration. A single firmness measurement per fruit was taken at the widest portion of the fruit halfway between the calyx and peduncle on the rosiest (ripest) portion of the fruit. In keeping with methods employed in previous studies (Messina and Jones 1990; Stoeckli et al. 2011), the apple skin was removed before taking the measurement. We used the pressure required to penetrate fruit for statistical analysis, which was calculated by dividing the penetrometer's measure of firmness by the cross‐sectional area of the probe used, resulting in a final measurement with units in kg per cm^2^. Early and late in the season, fruit firmness often was above and below, respectively, the measurement capabilities of the penetrometer. In such cases, fruit firmness was recorded as either the highest (1000 g for hawthorn and 13 kg for apple) or lowest (100 g for hawthorn and 0.5 kg for apple) possible value. Hawthorn fruit measurements were discontinued when all fruits’ penetration resistance were below the measurement capabilities of the penetrometer, which coincided with the time when essentially all fruit on trees was wrinkled, shrunken, or rotten*. Rhagoletis pomonella* do not oviposit into abscised fruit and rarely use fruit on trees that is beginning to rot (Dean and Chapman 1973; Boller and Prokopy 1976). Fruit measurements for early apples were discontinued when all fruit had abscised from trees and for late apples and ornamental hawthorn on 26 Sep 2013.

### Field eclosion time

To characterize the eclosion times of adult *R. pomonella* in the field at the Vancouver, Washington study site, we constructed 1.0 m length × 1.0 m width × 0.75 m height tents made of white tulle netting (mesh diameter ≤1‐mm) on the ground beneath the canopy of host trees to intercept eclosing flies. Within each tent, an unbaited 11.5 cm × 15.25 cm Pherocon^®^ No Bait AM Yellow Sticky Trap (Trece, Inc., Adair, OK) was hung from the inside‐top of the tent to attract and capture newly eclosing adult flies. The 27 tents used in the study were distributed among the same host trees used in the fruit ripeness study (Fig. 2). One tent was placed beneath each host tree with the exception that two tents were placed beneath one of the seven ornamental hawthorn trees. Tents were constructed beneath the portion of the tree's canopy with the most dense foliage and/or flowers on 27 May 2013. It was assumed that the highest number of fruit had fallen in those areas the previous year and therefore had the highest densities of overwintering *R. pomonella* pupae. Tents were checked three times per week from 30 May through 22 September 2013. Adult flies were removed from traps, counted, sexed, placed into 22 mL condiment cups (SOLO^®^ Cup Company, Lake Forest, IL), and frozen at −80°C within 24 h of collection. To account for potential shading effects on soil temperature associated with the tents, daily minimum and maximum soil temperatures were recorded using Digital Dual Sensor^®^ Thermometers (Forestry Suppliers, Inc., Jackson, MS) for three of the tents (1 in each of three areas denoted in Fig. 2). Each thermometer had two sensors attached to separate 1 m cables enabling soil temperatures to be recorded at a depth of 2 cm both inside and outside of the eclosion tents.

### Flight activity pattern

Adult flight activity was monitored at the Vancouver, WA site, to further assess the extent to which any observed differences in eclosion time coincided with variation in fruit maturation, as well as fly mating and oviposition, to quantify host‐associated allochronic isolation. Adult flight activity was measured by trapping flies in host tree canopies using Pherocon^®^ No Bait AM Yellow Sticky Traps. A single sticky trap affixed with a vial containing 15 g of ammonium carbonate was hung in the canopy of each of the 26 host trees used in the fruit ripeness and eclosion studies directly above the eclosion tent. The sticky traps in the tree canopies were monitored on the same days and in the same manner as those used to determine eclosion time in the ground tents.

### Larval infestation of fruit

To characterize the abundance of fly larvae during the field season, additional samples of fruit were collected from the canopy of each of the 26 study trees at the Vancouver, WA site, in the same manner as described above for the ripeness study. Collection schedules for host trees were black hawthorn fruits (12 July, 18 July, 12 August); early and late apple varieties (18 July, 31 July, and 14 August); and ornamental hawthorn fruits (6 and 26 September) (Table 1). Fruits were returned to the laboratory (Biology Department, Portland State University, Portland, OR) within 2 h of picking and placed into separate wire baskets made of 1.0 cm mesh‐size hardware cloth. The baskets were positioned above plastic collecting trays and held at a 15‐h/9‐h (light: dark) photoperiod at 22–23°C. Emerging larvae and puparia were counted and removed from the collecting trays every other day until none was found in the tray for an 8‐week period.

**Table 1:** Host species’ larval infestation times, as well as infestation rates based on numbers of larvae per fruit

Host	Date picked	# Fruit	# Larvae	# Larvae per fruit
Black haw	12‐Jul	7081	318	0.045
Black haw	18‐Jul	8152	910	0.112
Black haw	12‐Aug	942	57	0.061
Early apple	18‐Jul	40	280	7.000
Early apple	31‐Jul	63	554	8.794
Early apple	14‐Aug	30	162	5.400
Late apple	18‐Jul	99	236	2.384
Late apple	31‐Jul	141	360	2.553
Late apple	14‐Aug	147	437	2.973
Ornamental haw	6‐Sep	2294	248	0.108
Ornamental haw	26‐Sep	3516	1354	0.385

### Environmental data

Abiotic conditions might directly influence adult eclosion, resulting in a plastic, rather than genetic, basis for eclosion phenotypes. To assess environmental correlations with eclosion phenologies, we compiled ambient air temperature and precipitation data from the website Weather Underground (http://www.wunderground.com/history/ airport/KVUO/2013/3/25/MonthlyHistory.html?req_city= NA&req_state=NA&req statename=NA) for Pearson Field Airport (airport code KVUO), Vancouver, WA, located ~5 km from the study site.

### Data analysis

Individual measures of fruit firmness and size were divided by the respective maximums recorded in the study (firmness = 1000 g for hawthorn, 13 g for apple; size = 9.9 mm, 6.9 cm, 6.6 cm and 10.5 mm for black hawthorn, early apple, late apple, and ornamental hawthorn, respectively) to generate standardized metrics of relative ripeness for comparison among host species. Relative values for fruit firmness were then subtracted from 1 to produce measures of fruit softness that increased with time in accord with fruit ripening. Relative median fruit softness and mean fruit size data were then compared across sample dates for statistical significance between host species using paired *t*‐tests.

Eclosion time was analyzed using a linear mixed effects model, where “eclosion date” was considered as the response variable, “host” a fixed explanatory variable, and “individual tent” a random explanatory variable. Repeated‐measures analysis of variance (ANOVA) was performed to determine if mean eclosion times (± the standard error in days) varied among host populations. We used post hoc Tukey honestly significant difference multiple pairwise tests to determine which of the host populations differed significantly in eclosion time. One‐way ANOVA was conducted to determine whether mean dates of flight activity varied among host populations. To ensure that the assumptions of ANOVA were not violated, the normality of residuals was tested using qq‐plots and homoscedasticity using Brown–Forsythe tests. Pairwise estimates of allochronic isolation (AI) were calculated between host populations from the field eclosion curves and activity patterns of adult flies using the formula of Feder et al. (1993):

$$1-\left(\frac{\sum x_{i}y_{i}}{\sqrt{\sum x_{i}^{2}\cdot \sum y_{i}^{2}}}\right)\cdot 100,$$

where *x*_*i*_ and *y*_*i*_ are the proportions of the total numbers of flies present on host *x* or *y* on day *i*, assuming a mean adult life span of 30 days (Dean and Chapman 1973; Boller and Prokopy 1976). Newly eclosed adult *R. pomonella* are not sexually mature, but rather require 7–10 days for ovarian development (Dean and Chapman 1973). We therefore considered the period of adult sexual activity to be 10–30 days posteclosion. Spearman's rank correlation coefficients were used to test for significant relationships among cumulative fly eclosion, larval abundance, fruit ripeness (size and firmness), ambient air, and soil temperature (minimum, mean, and maximum), and rainfall. Daily eclosion was correlated with the preceding days’ temperature data. All data analyses were performed using R (R Core Team, 2013).

## Results

### Fruit ripeness

**Figure 3:**
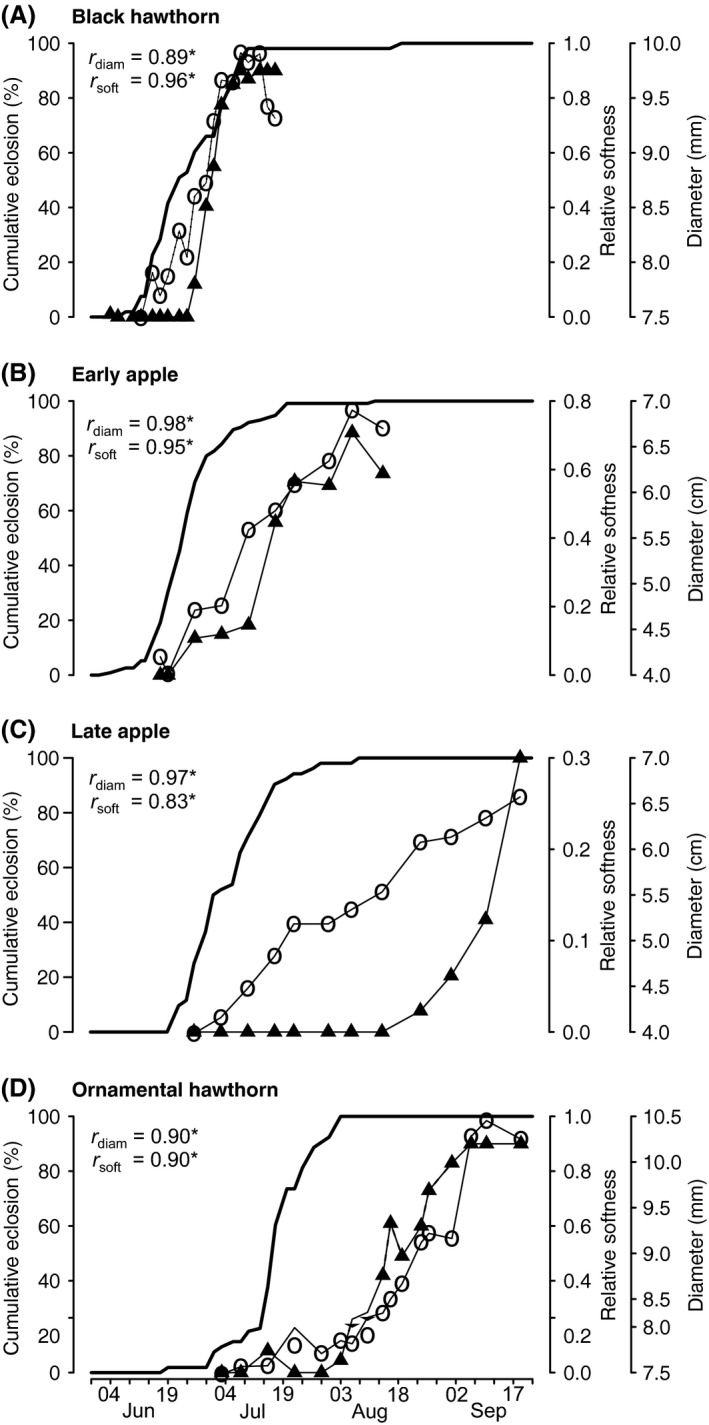
Cumulative adult eclosion (bold line), relative host fruit softness (filled triangles), and mean fruit diameter (open circles) plotted against time. Spearman's rank correlation coefficients are given for cumulative eclosion versus median fruit softness (*r*_soft_) and mean fruit diameter (*r*_diam_), where *P <* *0.0001. For standard errors and interquartile ranges of fruit ripening data see Figure S1.

**Figure 4:**
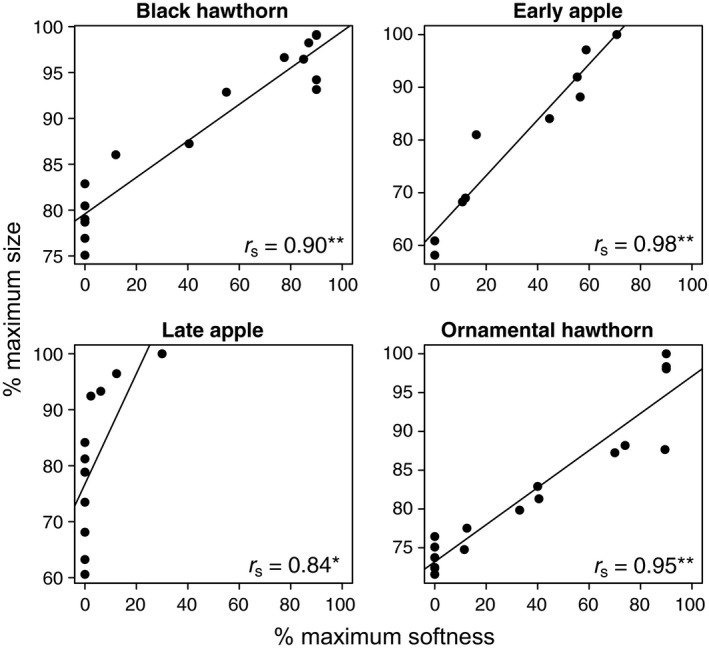
Percent maximum fruit softness and size were strongly correlated for each host species [Spearman's rank correlation coefficients (*r*_s_) and significance level (**P <* *0.0001; *P <* *0.001) shown in lower right of each graph].

The time when fruit ripened, as quantified by fruit softness and size, differed significantly among host species (Figs. 3 & S1, Table 2). However, comparisons of fruit size between early apple versus ornamental hawthorn, and late apple versus ornamental hawthorn, were not different and represent exceptions to the otherwise different fruiting phenologies (Figs. 3 & S1, Table 2). Softness and size were strongly positively correlated (Fig. 4); therefore, the term “ripeness” is henceforth used to imply both variables unless otherwise noted.

**Table 2:** Paired t‐tests comparing percent maximum (a) median fruit softness; and (b) mean size (diameter) through the sampling period. Host species with the earlier ripening time is listed first under “Host Pair”: BH = black hawthorn, EA = early apple, LA = late apple. Significant *P*‐values (*α *= 0.05) are in bold

Host pair	Mean % difference	*t*‐statistic	# Sample dates	*P*
(a) Median softness				
BH vs. EA	30.51	−2.19	6	**0.0398**
BH vs. LA	66.63	−3.62	4	**0.0181**
BH vs. OH	85.83	−20.60	3	**0.0012**
EA vs. LA	40.63	−4.78	8	**0.0010**
EA vs. OH	38.39	−3.30	4	**0.0229**
OH vs. LA	41.65	−3.38	8	**0.0059**
(b) Mean size				
BH vs. EA	18.90	9.71	6	**0.0001**
BH vs. LA	28.42	13.94	4	**0.0004**
BH vs. OH	24.52	16.64	3	**0.0018**
EA vs. LA	11.37	8.01	8	**<0.0001**
EA vs. OH	12.90	2.20	4	0.0575
LA vs. OH	1.76	0.97	8	0.1830

Black hawthorns ripened earliest, beginning in the last week of June and ending in the second week of July, when fruit became wrinkled and rotten. Early apple was chronologically second, beginning to ripen in the first week of July and concluding by the first week of August. The size of late apples increased steadily beginning in late June, but did not ripen until later in August, at which time they rapidly softened. Ornamental hawthorn fruit began increasing in size latest in the season, in the first week of August, but started softening prior to late apple fruits. Ornamental hawthorn fruit reached peak ripeness in the first week of September (Fig. 3).

### Field eclosion time

**Figure 5:**
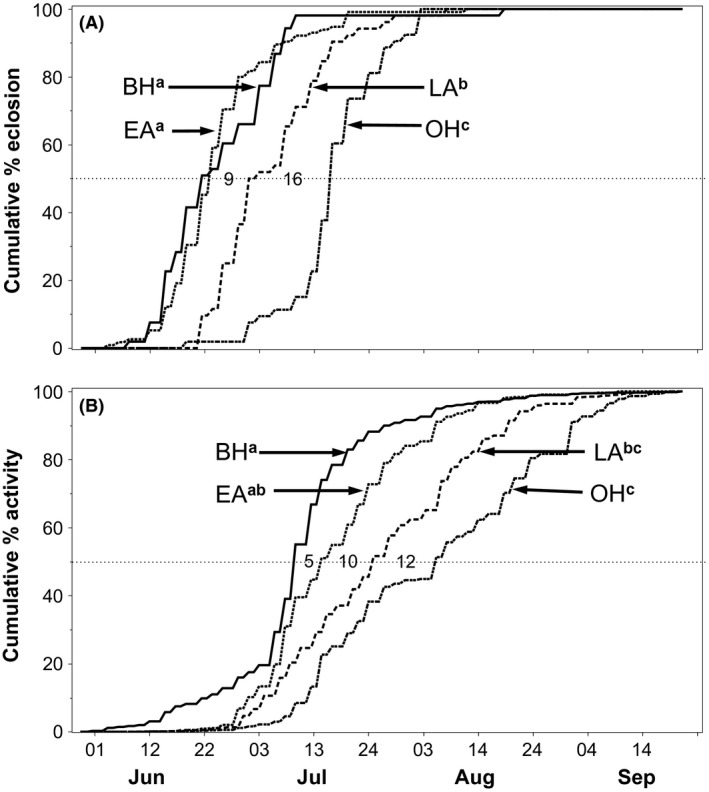
Cumulative (A) eclosion curves; and (B) flight activity times for black hawthorn (BH), early apple (EA), late apple (LA), and ornamental hawthorn (OH) flies at the Vancouver, WA study site. Sample sizes (numbers of flies) for generating the eclosion curves were *n *=* *53, 115, 52, and 53 for BH, EA, LA, and OH, respectively; and for activity curves *n *=* *1108, 539, 2074, and 873, respectively. Populations not sharing a superscript letter in common indicate a significant difference in mean eclosion or activity time (see Table 3 for significance levels). Numbers along the 50% stippled line of the *y*‐axis indicate the difference (in days) between median eclosion or activity times between host populations.

Adult eclosion times differed significantly among flies infesting black hawthorn, early‐fruiting apple, late‐fruiting apple, and ornamental hawthorn (*F*_3,14_ = 15.48, *P *<* *0.0001, Fig. 5). Post hoc analyses revealed that eclosion times differed significantly between all pairwise comparisons of host populations, with the exception of black hawthorn versus early apple flies (Table 3).

**Table 3:** Pairwise comparisons of (a) mean eclosion times; and (b) flight activity times between host‐associated fly populations. (BH = black hawthorn; EA = early apple; LA = late apple; OH= ornamental hawthorn). The population with the earlier phenology is listed first under “Hosts”. Difference = number of days between mean dates of (a) eclosion or (b) flight activity for indicated host comparisons. Significant *P*‐values (*α *= 0.05) are in bold

Hosts	Difference (Days)	SE	*z*‐value	*P*
(a) Eclosion				
BH ‐ EA	1.88	3.73	0.504	0.958
BH ‐ LA	12.04	3.45	3.493	**0.003**
BH ‐ OH	24.25	3.89	6.236	**<0.001**
EA ‐ LA	10.16	3.79	2.684	**0.036**
EA ‐ OH	22.37	4.19	5.337	**<0.001**
LA ‐ OH	12.21	3.94	3.099	**0.010**
(b) Activity				
BH ‐ EA	5.65	5.11	1.106	0.682
BH ‐ LA	15.43	3.83	4.026	**<0.001**
BH ‐ OH	26.00	4.02	6.462	**<0.001**
EA ‐ LA	9.79	5.28	1.855	0.243
EA ‐ OH	20.36	5.41	3.760	**0.001**
LA ‐ OH	10.57	4.24	2.496	0.059

The eclosion times of host populations generally matched the chronological sequence of fruit ripening. Adult flies were not captured in all the eclosion tents, although fruits from all trees in the study were infested with larvae. Mean date of eclosion for the 53 flies captured within the six black hawthorn eclosion tents producing adults was 23 June (±3.1 days SE); for the 115 flies captured from the three early apple tents, 26 June (±2.9); for the 52 flies captured from the five late apple tents, 5 July (±1.8); and for the 53 flies captured from four ornamental hawthorn tents, 17 July (±1.8) (Table S2). Estimates of premating allochronic isolation between black hawthorn‐associated flies and early apple‐, late apple‐, and ornamental hawthorn‐associated flies were 24.4%, 55.7%, and 92.6%, respectively. Isolation of early apple flies from late apple and ornamental hawthorn flies was estimated to be 43.9% and 87.1%, respectively, and between late apple and ornamental hawthorn flies, 59.3% (Table 4).

**Table 4:** Estimates of percent allochronic isolation among host populations as a result of (a) adult eclosion times, (b) flight activity times. Data collected from co‐occurring populations of *R. pomenella* in southwestern Washington State. (See text for calculations)

	Black hawthorn	Early apple	Late apple
(a) Eclosion			
Early apple	24.4%	–	–
Late apple	55.7%	43.9%	–
Ornamental hawthorn	92.6%	87.1%	59.3%
(b) Activity			
Early apple	14.7%	–	–
Late apple	31.3%	11.5%	–
Ornamental hawthorn	48.3%	36.6%	18.9%

Each host population displayed a significant positive relationship between eclosion time and both of the metrics of increasing fruit ripeness measured in the study (fruit softness: black hawthorn S = 1098.47, r_s,13_ = 0.96; early apple S = 233.86, r_s,7_ = 0.95; late apple S = 402.56, r_s,9_ = 0.83; ornamental hawthorn S = 1548.29, r_s,15_ = 0.90; increasing mean fruit diameter: black hawthorn S = 63.67, r_s,13_ = 0.89; early apple S = 2.53, r_s,7_ = 0.98; late apple S = 5.59, r_s,9_ = 0.97, *P *=* *0.002; ornamental hawthorn S = 78.10, r_s,15_ = 0.90; all *P *<* *0.0001 unless noted otherwise; Fig. 3). The differences between mean eclosion time and host fruit ripening increased from earlier to later fruiting host plants (Fig. 3).

Mean maximum soil temperatures were an average of 2.2°C cooler inside than outside tents during the day and 0.4°C warmer at night. Although these temperature differences were significant (Table S3), the lower maximum (during daytime) versus higher minimum (during nighttime) temperature differences resulted in roughly the same thermal accumulation and were consistent among hosts, thus were unlikely to have affected fly eclosion curves.

Tent soil temperature was not significantly correlated with cumulative eclosion time of host populations (black hawthorn S = 307.87, r_s,13_ = 0.45, *P = *0.09; early apple S = 47.48, r_s,7_ = 0.60, *P *=* *0.08; late apple S = 265.21, r_s,9_ = −0.21, *P *=* *0.54; ornamental hawthorn S = 1038.32, r_s,15_ = −0.27, *P = *0.29). However, ambient air temperature was related to cumulative eclosion for the black hawthorn (S = 199.28, r_s,13_ = 0.64, *P *=* *0.01) and early apple (S = 24.61, r_s,7_ = 0.79, *P *=* *0.01) populations, but not for the late apple (S = 116.01, r_s,9_ = 0.47, *P *=* *0.142) or ornamental hawthorn (S = 498.51, r_s,15_ = 0.39, *P *=* *0.12) populations. Only the black hawthorn population showed a slight, though significant relationship between cumulative adult eclosion and precipitation (black hawthorn S = 843.61, r_s,13_ = −0.51, *P *=* *0.05; early apple S = 181.28, r_s,7_ = −0.51, *P *=* *0.16; late apple S = 335.66, r_s,9_ = −0.53, *P *=* *0.10; ornamental hawthorn S = 546.47, r_s,15_ = 0.33, *P *=* *0.20).

### Flight activity pattern

**Figure 6:**
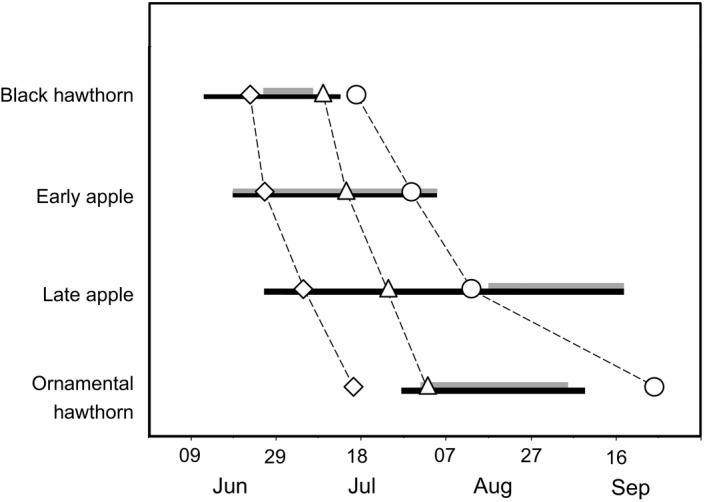
Mean dates of adult eclosion (diamonds) and flight activity (triangles), and maximum larval abundance (circles) for host populations at the Vancouver, WA site. Black and gray horizontal lines indicate ranges of dates when host fruits were most rapidly growing or softening, respectively.

Mean flight activity times of adult flies differed significantly among host populations (*F*_3,21_ = 13.01, *P *=* *5.07 × 10^−5^; Fig. 5) and chronologically matched host fruit ripening times (Fig. 6). Post hoc analyses revealed host‐associated differences in flight activity times in three of six pairwise comparisons (Table 3). Mean flight activity on black hawthorn trees was 10 July (±2.5 days SE); on early apple trees, 15 July (±3.3); for late apples, 25 July (±3.4); and for ornamental hawthorns, 3 August (±3.3). We found a consistent difference of 17–20 days between mean eclosion and flight activity times for all host populations.

Allochronic isolation due to differences in flight activity times was observed among hosts, but was less pronounced than estimates from eclosion time. Black hawthorn flies were 14.7%, 31.3%, and 48.3% allochronically isolated from early apple, late apple, and ornamental hawthorn flies, respectively, based on their flight activity patterns. Early apple flies were 11.5% and 36.6% isolated from late apple and ornamental hawthorn flies, respectively, and late apple flies were 18.9% allochronically isolated from ornamental hawthorn flies (Table 4).

### Larval abundance

Abundances of larvae in black hawthorn, early apple, late apple, and ornamental hawthorn fruit followed the sequence of fruit maturation, adult eclosion times, and the activity patterns of flies (Fig. 6). The highest densities of larvae reared from black hawthorn and ornamental hawthorn fruit were from 18 July (0.112 larvae per fruit) to 26 September (0.385 larvae per fruit), respectively. For early and late apple varieties, highest infestation levels occurred on 31 July (8.794 larvae per fruit) and 14 August (2.973 larvae per fruit), respectively (Table 1). While the time interval between peak adult eclosion and peak fly activity on trees was relatively constant across host populations (range 17 to 20 d), there was an increasing difference between activity time and maximum larval abundance between early‐ versus late‐fruiting hosts that was most pronounced for ornamental hawthorn (Figs. 3 and 6). One possible explanation for the difference is that eggs hatch and/or larvae develop more slowly in ornamental hawthorn fruits. It is also possible that ornamental hawthorn adults simply take longer to develop and reach sexual maturity and have a longer lifespan than early season flies, and therefore oviposit later than early season populations.

## Discussion

We found evidence that the life histories of *R. pomonella* populations infesting different host plants are temporally offset from one another in sympatry at a field site in Vancouver, WA, in accord with differences in the fruiting phenologies of their respective hosts. The differences in fruiting time generate partial allochronic mating isolation among fly populations that could contribute to the formation of host races in the PNW. The observed shifts in life‐history timing likely arose rapidly: an apple‐specialized race of *R. pomonella* was introduced to the PNW just 40–65 ya (Dowell 1988; Sim 2013). Black hawthorn and ornamental hawthorn are novel hosts whose ripening schedules are earlier and later, respectively, than apples, suggesting temporal changes in life history are not limited to shifting toward earlier phenologies, as seen for *R. pomonella* populations in the eastern USA.

It is still too early to conclude, however, that host races exist on black hawthorn, apple, and ornamental hawthorn in the PNW until additional studies show that genetic differences exist among these populations and confirm that the observed life‐history differences are heritable. In the eastern USA, eclosion time differences generating allochronic mating isolation between apple and downy hawthorn flies have been associated with genetic differences between the host races (Feder et al. 1993, 1997; Filchak et al. 2000; Egan et al. 2015). Thus, it is probable that similar genetic differences exist in the PNW, given that apple flies were introduced from the East. However, the eclosion differences reported in the current study were derived from flies experiencing nonuniform environmental conditions in the field. Consequently, rearing flies under standardized conditions in the laboratory could alter our conclusions. For instance, black hawthorn flies experience a prolonged period of elevated temperatures prior to winter and thus may require fewer days for development following the onset of warmer temperatures in the spring. Conversely, the opposite could be true for ornamental hawthorn flies. However, this explanation is unlikely, as previous studies have found concordance in eclosion times between noncontrolled field‐ and controlled laboratory‐reared flies, implying that the results obtained here reflect genetically based diapause differences in nature (Dambroski and Feder 2007; Lyons‐Sobaski and Berlocher 2009).

Even if a portion of the observed life‐history variation is subsequently shown to be plastic and environmentally induced; however, the differences would still generate allochronic mating isolation among host‐associated populations at the Vancouver site, facilitating the potential evolution of other ecological adaptations among the flies, including host discrimination and feeding performance (survivorship) differences. In this regard, apple and downy hawthorn flies in the eastern USA also display behavioral differences in host fruit odor discrimination (Linn et al. 2003; Dambroski et al. 2005; Forbes and Feder 2006). These differences are important because *R. pomonella* mate only on or near the fruit of their respective host plants (Prokopy et al. 1971, 1972). As a result, differences in host discrimination generate prezygotic reproductive isolation between apple and downy hawthorn flies (Feder et al. 1994). Similar behavioral differences in fruit volatile discrimination affecting host choice have been found among black hawthorn‐, apple‐, and ornamental hawthorn‐origin flies in the PNW (Linn et al. 2012; Sim et al. 2012), including at the Vancouver, WA site, consistent with the existence of host races in the region, as in the eastern USA.

In addition to the issues of genetics and environmental effects, verification of host races also requires showing that the pattern of allochronic isolation among black hawthorn, apple, and ornamental hawthorn flies at Vancouver, WA, in 2013 is consistent across years and other sites in the PNW. The black hawthorn stand examined in the current study was planted from nursery‐reared saplings between 2000 and 2006, as part of Burnt Bridge Creek Greenway (BBCG) riparian restoration project (R. Goughnour, pers. comm.). We found no other native black hawthorn growing in the immediate vicinity of the stand of over 30 trees at BBCG, aside from a single uninfested *C. suksdorfii* tree ~0.6 km away. Although the *C. douglasii* at BBCG were planted, their fruiting phenologies, fruit productivity, and infestation rates were very similar to those of other *C. douglasii* located at multiple nearby sites (e.g., Oak Island at Sauvie Island, OR [45° 42.8 N, 122° 49.1 W]; Washington State University Campus, Vancouver, WA [45° 44.0 N, 122° 37.6 W]; Legacy Salmon Creek Medical Center, Vancouver, WA [45° 43.2 N, 122° 39.0 W]; Springwater Corridor Trail near 99W in southeast Portland, OR [45° 27.7 N, 122° 38.1 W], M. Mattsson, pers. obs.). In addition, Tracewski et al. (1987), in a more coarse grained temporal analysis, reported peak *R. pomonella* flight activity and larval infestation of fruits to be 3–4 weeks earlier for black hawthorn than apple and ornamental hawthorn at a site in the Columbia River Gorge (St. Cloud Park, Skamania, WA) and 3–4 weeks earlier for apple than ornamental hawthorn at sites in the Portland and Vancouver area. In a six‐year study (1981–1986), AliNiazee and Westcott (1987) detected consistent differences in fly activity among apple, ornamental hawthorn, and in one case, black hawthorn populations across western Oregon. Thus, existing evidence suggests that the pattern we observed at the Vancouver site may hold to some degree at other locations, particularly with regard to ornamental hawthorn. However, local variability may also exist for black hawthorn and apple, requiring more fine‐grained seasonal analysis across the PNW to resolve if these hosts’ and associated fly phenologies are consistent throughout the region.

Another unresolved question concerns why estimates of allochronic isolation based on eclosion time were greater than those based on flight activity (Table 4). It is possible that posteclosion migration among hosts could reduce the effects of diapause timing on the temporal divergence of fly populations. In this regard, early‐ and late‐fruiting apple varieties could serve as a conduit for gene flow among hosts and decrease the consequences of divergent diapause selection on allochronic mating isolation. It is also possible that the increased temporal overlap in flight activity was a consequence of the methods we used to trap flies in trees. In particular, the sticky traps, being yellow in color and baited with ammonium carbonate, are generally attractive to *R. pomonella* (Moericke et al. 1975; Rull and Prokopy 2000; Pelz‐Stelinski et al. 2005; Yee and Goughnour 2011). Adult flies often forage for food across relatively large distances (0.6 km or more; Bourne et al. 1934; Maxwell and Parsons 1968; Feder et al. 1994) before returning to host trees to mate and oviposit upon reaching sexual maturity. Thus, the baited yellow sticky traps, which are chemotactically and visually attractive to western *R. pomonella* (Yee and Goughnour 2011), may have resulted in higher proportions of non‐natal flies being trapped in trees than is normally the case. Sampling of flies by other means (i.e., by aspiration directly from host plants) is therefore needed to help discern the cause for the increased temporal overlap of fly populations based on flight activity. We note, however, that levels of AI derived from adult flight activity are nevertheless comparable to those estimated between apple and downy hawthorn flies in the eastern USA (Feder et al. 1994; Feder 1998) and, thus, may be sufficient to facilitate host race formation in the PNW.

In conclusion, we documented that seasonal differences in eclosion, flight activity, and larval abundance times exist among host‐associated populations of *R. pomonella* at a field site in Vancouver, Washington, USA. These life‐history differences generate allochronic isolation, similar to the apple and downy host races of the fly in the eastern USA. However, in contrast to the eastern USA, flies in the PNW appear to have switched from apple to two novel hawthorn species within the last 40–65 years and shifted their life histories to exploit later, as well as earlier, fruiting hosts. Additional studies are required (1) to confirm the genetic basis for the eclosion time differences; (2) to determine how environmental cues such as temperature, moisture, and photoperiod, act in concert with gene expression to affect the onset, cessation, and – ultimately – the length of diapause in *R. pomonella* (Prokopy 1968; Filchak et al. 1999); and (3) to discern the exact stage(s) in the life cycle in which environmental cues influence the diapause schedule of the fly. Previous work indicates that larval and pupal stages of *R. pomonella* respond to temperature and photoperiod cues, whereas the egg and adult stages do not (Prokopy 1968; Feder et al. 1997). Diapause life‐history adaptation to match host phenology is common in phytophagous insects (Tauber et al. 1986). Consequently, diapause life history may often rapidly evolve to ecologically adapt phytophagous insects to novel or changing temporal host resources (Funk et al. 2002; Bradshaw and Holzapfel 2008; Wadsworth et al. 2013). Such shifts can have repercussions for the timing of reproduction that result in allochronic isolation among host‐associated populations that can potentially initiate speciation regardless of geographic context (in the face of gene flow or in allopatry in its absence). Given that there are more phytophagous insect specialists than any other life‐form (Bush 1975; Price 1977), diapause life‐history adaptation may be a frequent contributor to the great diversity of life on Earth.

## Conflict of Interest

None declared.

## Supporting information


**Figure S1** Mean diameter (±SE) and firmness (± interquartile range) for hawthorn and apple fruit at the Vancouver, WA sites through time.

**Table S1** Dates of fruit collection for measuring fruit softness (F), size (S), and larval abundance (L).

**Table S2** Mean dates of eclosion (± standard error in days) for individual eclosion tents as well as host population (BH = black hawthorn; EA = early apple; LA = late apple; OH = ornamental hawthorn), and the mean of the individual tent means (Population Ordinal Mean) for each population. Ordinal date 165 = 14–Jun.

**Table S3** Eclosion tents dampened fluctuations in soil temperatures within the enclosure.

## References

[ece31826-bib-0001] Abrahamson, W. G. , J. M. Brown , S. K. Roth , D. V. Sumerford , J. D. Horner , M. D. Hess , et al. 1994 Gallmaker speciation: an assessment of the roles of host‐plant characters, phenology, gallmaker competition, and natural enemies Pp. 208–222 *in* Price P., Mattson W. and Baranchilov Y., eds. Gall‐forming insects: general technical report NC‐174. USDA Forest Service, North Central Experimental Station.

[ece31826-bib-0002] AliNiazee, M. T. , and R. L. Penrose . 1981 Apple maggot in Oregon: a possible new threat to the Northwest apple industry. Bull. Entom. Soc. Am. 27:245–246.

[ece31826-bib-0003] AliNiazee, M. T. , and R. L. Westcott . 1986 Distribution of the apple maggot, *Rhagoletis pomonella* (Diptera: Tephritidae) in Oregon. J. Entomol. Soc. BC. 83:54–56.

[ece31826-bib-0004] AliNiazee, M. T. , and R. L. Westcott . 1987 Flight period and seasonal development of the apple maggot, *Rhagoletis pomonella* (Walsh) (Diptera: Tephritidae) Oregon. Ann. Entomol. Soc. Am. 80:823–828.

[ece31826-bib-0005] Berlocher, S. H. , and J. L. Feder . 2002 Sympatric speciation in phytophagous insects: moving beyond controversy? Annu. Rev. Entomol. 47:773–815. 11729091 10.1146/annurev.ento.47.091201.145312

[ece31826-bib-0006] Boller, E. F. , and R. J. Prokopy . 1976 Bionomics and management of *Rhagoletis* . Ann. Rev. of Entomol. 21:223–246.

[ece31826-bib-0007] Bourne, A. I. , W. H. Thies , and F. R. Shaw . 1934 Some observation of long distance dispersal of apple maggot flies. J. Econ. Entomol. 27:352–355.

[ece31826-bib-0008] Bradshaw, W. E. , and C. M. Holzapfel . 2008 Genetic response to rapid climate change: it's seasonal timing that matters. Mol. Ecol. 17:157–166. 17850269 10.1111/j.1365-294X.2007.03509.x

[ece31826-bib-0009] Bush, G. L. 1966 The taxonomy, cytology and evolution of the genus Rhagoletis in North America (Diptera:Tephritidae). Museum of Comparative Zoology, Cambridge, MA.

[ece31826-bib-0010] Bush, G. L. 1969 Sympatric host race formation and speciation in frugivorous flies of the genus *Rhagoletis* (Diptera: Tephritidae). Evolution 23:237–251. 10.1111/j.1558-5646.1969.tb03508.x 28562891

[ece31826-bib-0011] Bush, G. L. 1975 Sympatric speciation in phytophagous parasitic insects Pp. 187–206 *in* Price P. W., ed. Evolutionary strategies of parasitic insects and mites. Plenum, New York.

[ece31826-bib-0012] Bush, G. L. 1994 Host race formation and sympatric speciation in Rhagoletis fruit flies (Diptera: Tephritidae). Psyche 99:335–357.

[ece31826-bib-0013] Dambroski, H. R. , and J. L. Feder . 2007 Host plant and latitude‐related diapause variation in *Rhagoletis pomonella*: a test for multifaceted life history adaptation on different stages of diapause development. Evolution 20:2101–2112. 10.1111/j.1420-9101.2007.01435.x 17956381

[ece31826-bib-0014] Dambroski, H. R. , C. Jr Linn , S. H. Berlocher , A. A. Forbes , W. Roelofs , and J. L. Feder . 2005 The genetic basis for fruit odor discrimination in *Rhagoletis* flies and its significance for sympatric host shifts. Evolution 59:1953–1964. 16261733

[ece31826-bib-0015] Dean, R. W. , and P. J. Chapman . 1973 Bionomics of the apple maggot in eastern New York. Search Agric. Entomol. Geneva 3:1–64.

[ece31826-bib-0016] Denlinger, D. L. 2002 Regulation of diapause. Annu. Rev. Entomol. 47:93–122. 11729070 10.1146/annurev.ento.47.091201.145137

[ece31826-bib-0017] Diehl, S. R. , and G. L. Bush . 1984 An evolutionary and applied perspective of insect biotypes. Ann. Rev. Entomol. 29:471–504.

[ece31826-bib-0018] Dowell, R. V. 1988 History of apple maggot in the western United States p. 3 *in* Dowell R. V., Wilson L. T. and Jones V. P., eds. Apple maggot in the West. University of California Division of Agriculture and Natural Resources, Oakland, CA.

[ece31826-bib-0019] Drès, M. , and J. Mallet . 2002 Host races in plant‐feeding insects and their importance in sympatric speciation. Philos. Trans. R. Soc. Lond. 357:471–492. 12028786 10.1098/rstb.2002.1059 PMC1692958

[ece31826-bib-0020] Egan, S. P. , G. R. Ragland , L. Assour , T. H. Q. Powell , G. R. Hood , S. Emrich , et al. 2015 Experimental evidence of genome‐wide impact of ecological selection during early stages of speciation‐with‐gene‐flow. Ecol. Lett. 18:817–825. 26077935 10.1111/ele.12460 PMC4744793

[ece31826-bib-0021] Feder, J. L. 1995 The effects of parasitoids on sympatric host races of *Rhagoletis pomonella* (Diptera: Tephritidae). Ecology 76:801–813.

[ece31826-bib-0022] Feder, J. L. 1998 The apple maggot fly, *Rhagoletis pomonella*: flies in the face of conventional wisdom about speciation? Pp. 130–144 *in* Howard D. J. and Berlocher S. H., eds. Endless forms: species and speciation. Oxford Univ. Press, New York.

[ece31826-bib-0023] Feder, J. L. , C. A. Chilcote , and G. L. Bush . 1988 Genetic differentiation between sympatric host races of the apple maggot fly *Rhagoletis pomonella* . Nature 336:61–64.

[ece31826-bib-0024] Feder, J. L. , T. A. Hunt , and G. L. Bush . 1993 The effects of climate, host plant phenology and host fidelity on the genetics of apple and hawthorn infesting races of *Rhagoletis pomonella* . Entomol. Exp. Appl. 69:117–135.

[ece31826-bib-0025] Feder, J. L. , S. B. Opp , B. Wlazlo , K. Reynolds , W. Go , and S. Spisak . 1994 Host fidelity is an effective premating barrier between sympatric races of the apple maggot fly. Proc. Natl Acad. Sci. USA 91:7990–7994. 11607491 10.1073/pnas.91.17.7990 PMC44530

[ece31826-bib-0026] Feder, J. L. , K. Reynolds , W. Go , and E. C. Wang . 1995 Intra‐ and interspecific competition and host race formation in the apple maggot fly (Diptera: Tephritidae). Oecologia 101:416–425. 10.1007/BF00329420 28306956

[ece31826-bib-0027] Feder, J. L. , J. B. Roethele , B. Wlazlo , and S. H. Berlocher . 1997 Selective maintenance of allozyme differences among sympatric host races of the apple maggot fly. Proc. Natl Acad. Sci. USA 94:11417–11421. 11038585 10.1073/pnas.94.21.11417 PMC23485

[ece31826-bib-0028] Filchak, K. E. , J. L. Feder , J. B. Roethele , and U. Stoltz . 1999 A field test for host‐plant dependent selection on larvae of the apple maggot fly, *Rhagoletis pomonella* . Evolution 53:187–200. 10.1111/j.1558-5646.1999.tb05344.x 28565200

[ece31826-bib-0029] Filchak, K. E. , J. B. Roethele , and J. L. Feder . 2000 Natural selection and sympatric divergence in the apple maggot *Rhagoletis pomonella* . Nature 407:739–742. 11048719 10.1038/35037578

[ece31826-bib-0030] Forbes, A. A. , and J. L. Feder . 2006 Divergent preferences of *Rhagoletis pomonella* host races for olfactory and visual cues. Entomol. Exp. Appl. 119:121–127.

[ece31826-bib-0031] Funk, D. J. , K. E. Filchak , and J. L. Feder . 2002 Herbivorous insects: model systems for the comparative study of speciation ecology. Genetica 116:251–267. 12555783

[ece31826-bib-0032] Groman, J. D. , and O. Pellmyr . 2000 Rapid evolution and specialization following host colonization in a yucca moth. J. Evol. Biol. 13:223–236.

[ece31826-bib-0033] Harrison, R. G. 1985 Barriers to gene exchange between closely related cricket species. II. Life cycle variation and temporal isolation. Evolution 39:244–259. 10.1111/j.1558-5646.1985.tb05664.x 28564221

[ece31826-bib-0034] Hood, G. R. , W. Yee , R. B. Goughnour , S. B. Sim , S. P. Egan , T. Arcella , et al. 2013 The geographic distribution of *Rhagoletis pomonella* (Diptera: Tephritidae) in the western United States: introduced species or native population? Ann. Entomol. Soc. Am. 106:59–65.

[ece31826-bib-0035] Horner, J. D. , T. P. Craig , and J. K. Itami . 1999 The influence of oviposition phenology on survival in host races of *Eurosta solidaginis* . Entomol. Exp. Appl. 93:121–129.

[ece31826-bib-0036] How, S. T. , W. G. Abrahamson , and T. P. Craig . 1993 Role of host plant phenology in host use by *Eurosta solidaginis* (Diptera: Tephritidae) on *Solidago* (Compositae). Environ. Entomol. 22:388–396.

[ece31826-bib-0037] Linn, C. E. Jr , J. L. Feder , S. Nojima , H. R. Dambroski , S. H. Berlocher , and W. Roelofs . 2003 Fruit odor discrimination and sympatric host race formation in *Rhagoletis* . Proc. Natl Acad. Sci. 100:11490–11493. 14504399 10.1073/pnas.1635049100 PMC208785

[ece31826-bib-0038] Linn, C. E. Jr , W. L. Yee , S. B. Sim , D. H. Cha , T. H. Q. Powell , R. B. Goughnour , et al. 2012 Behavioral evidence for fruit odor discrimination and sympatric host races of *Rhagoletis pomonella* flies in the western United States. Evolution 66:2632–2641. 10.1111/j.1558-5646.2012.01719.x 23106724

[ece31826-bib-0039] Lyons‐Sobaski, S. , and S. H. Berlocher . 2009 Life history phenology differences between southern and northern populations of the apple maggot fly, *Rhagoletis pomonella* . Entomol. Exp. Appl. 130:149–159.

[ece31826-bib-0040] Maxwell, C. W. , and E. C. Parsons . 1968 The recapture of marked apple maggot adults in several orchards from one release point. J. Econ. Entomol. 61:1157–1159.

[ece31826-bib-0041] McPheron, B. A. 1987 The population genetics of the infestation of the western United States by the Apple Maggot, Rhagoletis pomonella (Walsh) (Diptera: Tephritidae). Ph.D. Dissertation. Univ. of Illinois, Urbana. pp. 107–150.

[ece31826-bib-0042] McPheron, B. A. , D. C. Smith , and S. H. Berlocher . 1988 Genetic differences between host races of *Rhagoletis pomonella* . Nature 336:64–66.

[ece31826-bib-0044] Messina, F. J. , and V. P. Jones . 1990 Relationship between fruit phenology and infestation by the apple maggot (Diptera: Tephritidae) Utah. Ann. Entomol. Soc. Am. 83:742–752.

[ece31826-bib-0045] Moericke, V. , R. J. Prokopy , S. Berlocher , and G. L. Bush . 1975 Visual stimuli eliciting attraction of *Rhagoletis pomonella* flies to trees. Entomol. Exp. Appl. 18:497–507.

[ece31826-bib-0046] Nosil, P. 2012 Ecological speciation. 1st edn Oxford Univ. Press, Oxford.

[ece31826-bib-0047] Pelz‐Stelinski, K. S. , L. J. Gut , L. L. Stelinski , O. E. Liburd , and R. Isaacs . 2005 Captures of *Rhagoletis mendax* and *R. cingulate* (Diptera:Tephritidae) on sticky traps are influenced by adjacent host fruit and fruit juice concentrates. Environ. Entomol. 34:1013–1018.

[ece31826-bib-0048] Price, P. W. 1977 General concepts on the evolutionary biology of parasites. Evolution 31:405–420. 10.1111/j.1558-5646.1977.tb01021.x 28563224

[ece31826-bib-0049] Prokopy, R. J. 1968 Influence of photoperiod, temperature, and food on initiation of diapause in the apple maggot. Can. Entomol. 100:318–329.

[ece31826-bib-0050] Prokopy, R. J. , E. W. Bennett , and G. L. Bush . 1971 Mating behavior in *Rhagoletis pomonella* (Diptera: Tephritidae) I Site of assembly. Can. Entomol. 103:1405–1409.

[ece31826-bib-0051] Prokopy, R. J. , E. W. Bennett , and G. L. Bush . 1972 Mating behavior in *Rhagoletis pomonella* (Diptera: Tephritidae) II Temporal organization. Can. Entomol. 104:97–104.

[ece31826-bib-0052] R Core Team . 2013 R: A language and environment for statistical computing. R Foundation for Statistical Computing, Vienna, Austria URL http://www.R-project.org/).

[ece31826-bib-0053] Rull, J. , and R. J. Prokopy . 2000 Attraction of apple maggot flies, *Rhagoletis pomonella* (Diptera:Tephritidae) of different physiological states to odour‐baited traps in the presence and absence of food. Bull. Entomol. Res. 90:77–88. 10948367

[ece31826-bib-0054] Rundle, H. D. , and P. Nosil . 2005 Ecological speciation. Ecol. Lett. 8:336–352.

[ece31826-bib-0055] Schluter, D. 2001 Ecology and the origin of species. Trends Ecol. Evol. 16:372–380. 11403870 10.1016/s0169-5347(01)02198-x

[ece31826-bib-0501] Sharp, J. L. 1978 Tethered flight of apple maggot flies. Fla. Entomol. 61:199‐200.

[ece31826-bib-0056] Sim, S. 2013 The frontier of ecological speciation: investigating western populations of Rhagoletis pomonella. PhD dissertation. University of Notre Dame, Notre Dame, IN.

[ece31826-bib-0057] Sim, S. B. , M. Mattsson , J. L. Feder , D. H. Cha , W. L. Yee , R. B. Goughnour , et al. 2012 A field test for host fruit odour discrimination and avoidance behavior for *Rhagoletis pomonella* flies in the western United States. J. Evol. Biol. 25:961–971. 22435643 10.1111/j.1420-9101.2012.02489.x

[ece31826-bib-0058] Smith, D. C. 1988 Heritable divergence of *Rhagoletis pomonella* host races by seasonal asynchrony. Nature 336:66–67.

[ece31826-bib-0059] Stoeckli, S. , K. Mody , and S. Dorn . 2011 Association between herbivore resistance and fruit quality in apple. HortScience 46:12–15.

[ece31826-bib-0060] Tauber, M. J. , C. A. Tauber , and S. Masaki . 1986 Seasonal adaptations of insects. Oxford Univ. Press, New York.

[ece31826-bib-0061] Tracewski, K. T. , J. F. Brunner , S. C. Hoyt , and S. R. Dewey . 1987 Occurrence of *Rhagoletis pomonella* (Walsh) in hawthorns, Crataegus, of the Pacific Northwest. Melanderia 45:19–25.

[ece31826-bib-0043] U.S. Department of Agriculture . 2013 Washington Annual Agricultural Bulletin. *in* Mertz C., Koong D., and Anderson S., eds. National Agricultural Statistics Service Northwest Regional Field Office http://www.nass.usda.gov/Statistics_by_State/Washington/Publications/Annual_Statistical_Bulletin/

[ece31826-bib-0062] Via, S. 1999 Reproductive isolation between sympatric races of pea aphids. I. gene flow restriction and habitat choice. Evolution 53:1446–1457. 10.1111/j.1558-5646.1999.tb05409.x 28565574

[ece31826-bib-0063] Via, S. , A. C. Bouck , and S. Skillman . 2000 Reproductive isolation among divergent races of pea aphids on two hosts. II. Selection against migrants and hybrids in the parental environments. Evolution 54:1626–1637. 11108590 10.1111/j.0014-3820.2000.tb00707.x

[ece31826-bib-0064] Wadsworth, C. B. , W. A. Jr Woods , D. A. Hahn , and E. B. Dopman . 2013 One phase of the dormancy developmental pathway is critical for the evolution of insect seasonality. J. Evol. Biol. 26:2359–2368. 24016035 10.1111/jeb.12227

[ece31826-bib-0065] Walsh, B. D. 1867 The apple‐worm and the apple‐maggot. J. Hort. 2:338–343.

[ece31826-bib-0066] Wood, T. K. , K. J. Tilmon , A. B. Shantz , C. K. Harris , and J. Pesek . 1999 The role of host‐plant fidelity in initiating insect race formation. Evol. Ecol. Res. 1:317–332.

[ece31826-bib-0067] Yee, W. L. 2008 Host plant use by apple maggot, western cherry fruit fly, and other Rhagoletis species (Diptera:Tephritidae) in central Washington state. Pan‐Pac. Entomol. 84:163–178.

[ece31826-bib-0068] Yee, W. L. , and R. B. Goughnour . 2011 Differential captures of *Rhagoletis pomonella* (Diptera:Tephritidae) on four fluorescent yellow rectangle traps. Fla. Entomol. 94:998–1009.

[ece31826-bib-0069] Yee, W. L. , M. W. Klaus , D. H. Cha , C. E. Jr Linn , R. B. Goughnour , and J. L. Feder . 2012 Abundance of apple maggot, *Rhagoletis pomonella*, across different areas in central Washington, with special reference to black‐fruited hawthorns. J. Insect Sci. 1:1–14. 10.1673/031.012.12401 PMC3633246 23451979

