# Supplementary figures and images for: Rapid and repeatable shifts in life‐history timing of Rhagoletis pomonella (Diptera: Tephritidae) following colonization of novel host plants in the Pacific Northwestern United States
Source: Ecol Evol. 2015 Nov 26;5(24):5823–37. doi: 10.1002/ece3.1826 (PMC4717349; doi:10.1002/ece3.1826)

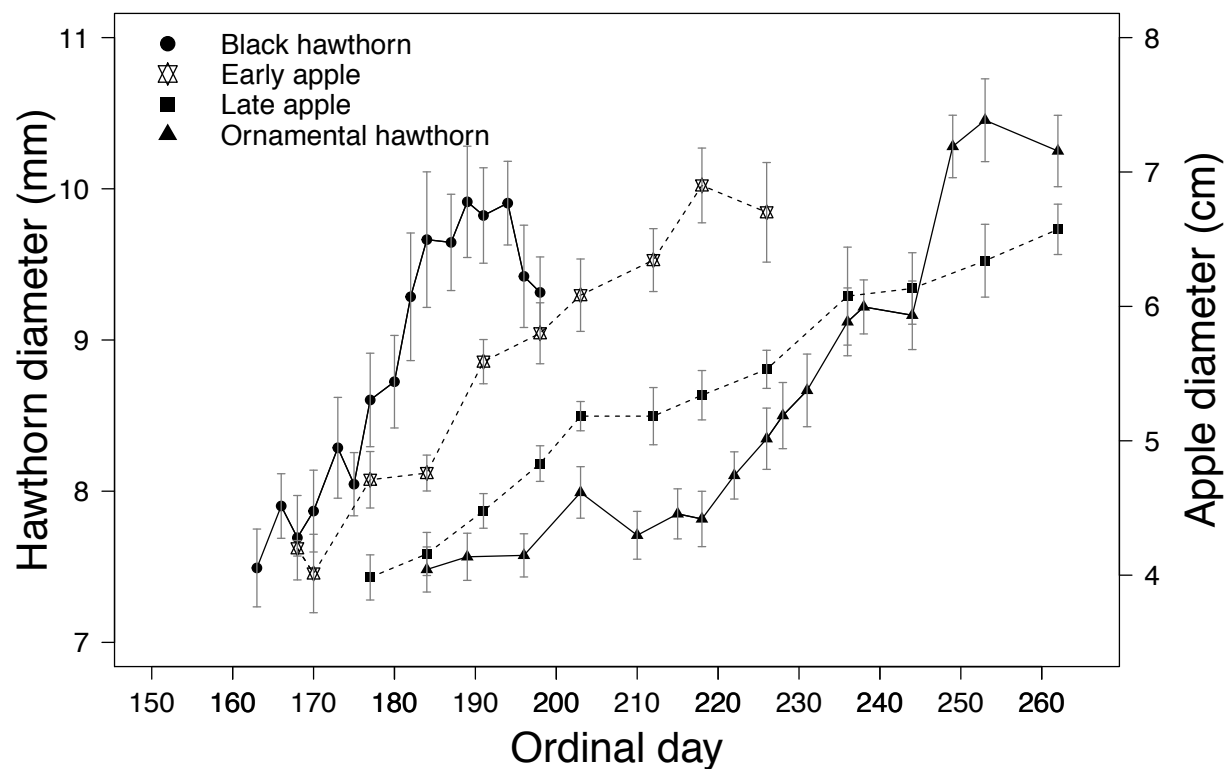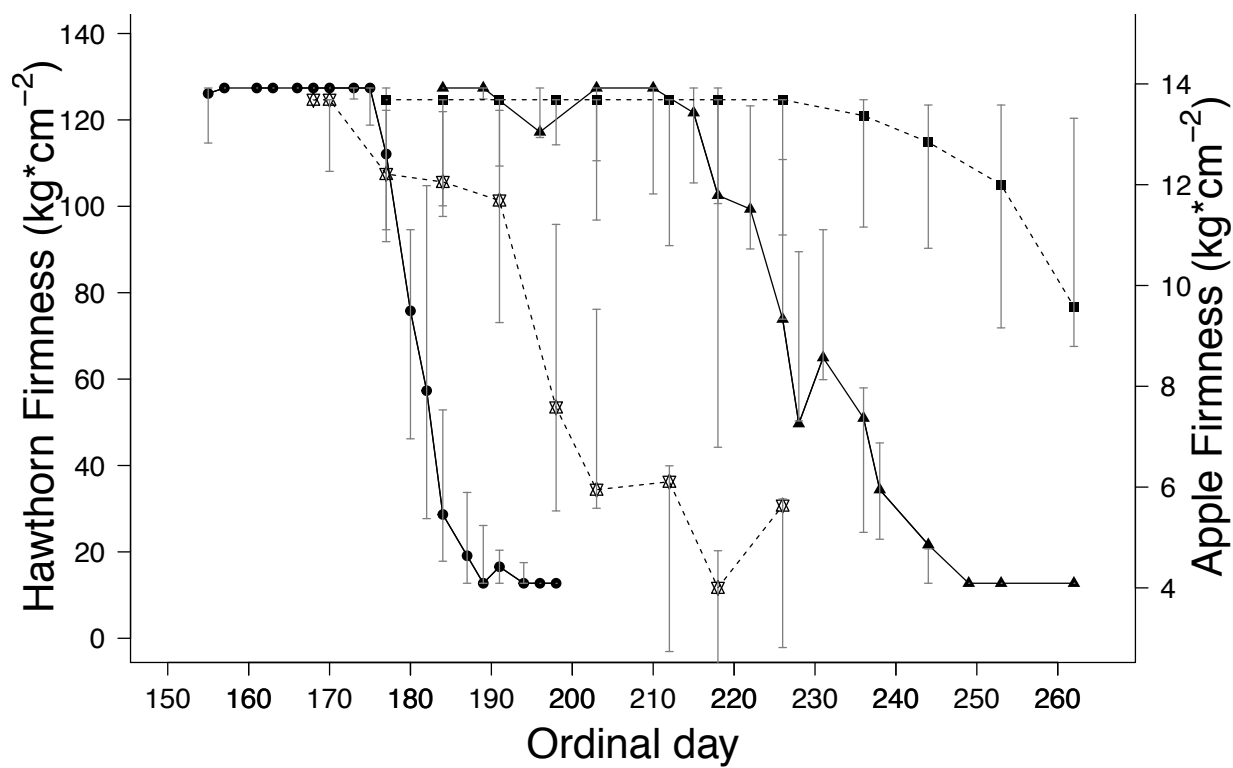

Supplement: Supplementary file 1 — Figure S1 Mean diameter (±SE) and firmness (± interquartile range) for hawthorn and apple fruit at the Vancouver, WA sites through time. [file ECE3-5-5823-s001.pdf]
