# Supplementary material for: Rapid and repeatable shifts in life‐history timing of Rhagoletis pomonella (Diptera: Tephritidae) following colonization of novel host plants in the Pacific Northwestern United States
Source: Ecol Evol. 2015 Nov 26;5(24):5823–37. doi: 10.1002/ece3.1826 (PMC4717349; doi:10.1002/ece3.1826)
Supplement: Supplementary file 2 — Table S1 Dates of fruit collection for measuring fruit softness (F), size (S), and larval abundance (L). [file ECE3-5-5823-s002.docx]

| Table S1 Dates of fruit collection for measuring fruit softness (F), size (S), and larval abundance (L). | | | | |
| --- | --- | --- | --- | --- |
|  |  |  |  |  |
| Date | Black haw | Early apple | Late apple | Orn. haw |
| 12-Jun-13 | F, S |  |  |  |
| 15-Jun-13 | F, S |  |  |  |
| 17-Jun-13 | F, S |  |  |  |
| 19-Jun-13 | F, S |  |  |  |
| 22-Jun-13 | F, S |  |  |  |
| 24-Jun-13 | F, S |  |  |  |
| 26-Jun-13 | F, S | F, S | F, S |  |
| 29-Jun-13 | F, S |  |  |  |
| 1-Jul-13 | F, S |  |  |  |
| 3-Jul-13 | F, S | F, S | F, S | F, S |
| 6-Jul-13 | F, S |  |  |  |
| 8-Jul-13 | F, S |  |  | F, S |
| 10-Jul-13 | F, S | F, S | F, S |  |
| 12-Jul-13 | F, S, L | F, S | F, S |  |
| 13-Jul-13 | F, S |  |  |  |
| 15-Jul-13 | F, S |  |  | F, S |
| 17-Jul-13 | F, S | F, S | F,S |  |
| 18-Jul-13 | L | F, S, L | F, S, L |  |
| 22-Jul-13 |  | F,S | F,S | F, S |
| 29-Jul-13 |  |  |  | F, S |
| 31-Jul-13 |  | F, S, L | F, S, L |  |
| 3-Aug-13 |  |  |  | F, S |
| 6-Aug-13 |  | F, S | F, S | F, S |
| 10-Aug-13 |  |  |  | F, S |
| 12-Aug-13 | L |  |  | F, S |
| 14-Aug-13 |  | F, S, L | F, S, L | F, S |
| 16-Aug-13 |  |  |  | F, S |
| 19-Aug-13 |  |  |  | F, S |
| 24-Aug-13 |  |  | F, S | F, S |
| 26-Aug-13 |  |  |  | F, S |
| 1-Sep-13 |  |  | F, S | F, S |
| 6-Sep-13 |  |  |  | F, S, L |
| 10-Sep-13 |  |  | F, S | F, S |
| 19-Sep-13 |  |  | F, S | F, S |
| 26-Sep-13 |  |  |  | L |
