# Supplementary material for: Rapid and repeatable shifts in life‐history timing of Rhagoletis pomonella (Diptera: Tephritidae) following colonization of novel host plants in the Pacific Northwestern United States
Source: Ecol Evol. 2015 Nov 26;5(24):5823–37. doi: 10.1002/ece3.1826 (PMC4717349; doi:10.1002/ece3.1826)
Supplement: Supplementary file 3 — Table S2 Mean dates of eclosion (± standard error in days) for individual eclosion tents as well as host population (BH = black hawthorn; EA = early apple; LA = late apple; OH = ornamental hawthorn), and the mean of the individual tent means (Population Ordinal Mean) for each population. Ordinal date 165 = 14–Jun. [file ECE3-5-5823-s003.docx]

| **Table S2** Mean dates of eclosion (+ standard error in days) for individual eclosion tents as well as host population (BH = black hawthorn; EA = early apple; LA = late apple; OH = ornamental hawthorn), and the mean of the individual tent means (Population Ordinal Mean) for each population. Ordinal date 165 = 14–Jun. | | | | | | | |
| --- | --- | --- | --- | --- | --- | --- | --- |
| **Host** | **Tent ID** | **# Flies** | **Mean**  **Ordinal Date** | **Tent s.e.** | **Median Ordinal Date** | **Population Ordinal Mean** | **Population**  **s.e.** |
| BH | DGBH 02 | 22 | 180.68 | 3.07 | 181.50 | 174.12 | 3.14 |
| BH | DGBH 03 | 17 | 177.24 | 2.06 | 176.50 | – | – |
| BH | DGBH 05 | 4 | 168.50 | 0.66 | 168.50 | – | – |
| BH | DGBH 06 | 3 | 167.00 | 1.60 | 167.50 | – | – |
| BH | DGBH 07 | 2 | 184.50 | 3.10 | 184.00 | – | – |
| BH | DGBH 09 | 5 | 166.80 | 2.24 | 180.00 | – | – |
|  |  |  |  |  |  |  |  |
| EA | BOB AP | 3 | 180.67 | 8.36 | 172.50 | 177.41 | 2.95 |
| EA | WILK AP 01 | 49 | 171.53 | 1.10 | 169.50 | – | – |
| EA | WILK AP 04 | 63 | 180.05 | 1.25 | 176.50 | – | – |
|  |  |  |  |  |  |  |  |
| LA | WILK AP 02 | 22 | 182.73 | 1.88 | 179.50 | 186.47 | 1.81 |
| LA | WILK AP 03 | 7 | 187.14 | 1.83 | 188.50 | – | – |
| LA | WILK AP 06 | 4 | 186.25 | 4.31 | 188.50 | – | – |
| LA | WILK AP 07 | 3 | 183.33 | 3.29 | 181.50 | – | – |
| LA | WILK AP 08 | 16 | 192.88 | 3.05 | 191.00 | – | – |
|  |  |  |  |  |  |  |  |
| OH | DISC OH 04 | 1 | 194.00 |  | 193.50 | 198.36 | 1.79 |
| OH | ALKI OH 01 | 44 | 198.45 | 1.41 | 197.50 | – | – |
| OH | ALKI OH 02 | 4 | 198.25 | 2.01 | 196.50 | – | – |
| OH | BBC OH | 4 | 202.75 | 2.86 | 204.00 | – | – |
