# Supplementary material for: Rapid and repeatable shifts in life‐history timing of Rhagoletis pomonella (Diptera: Tephritidae) following colonization of novel host plants in the Pacific Northwestern United States
Source: Ecol Evol. 2015 Nov 26;5(24):5823–37. doi: 10.1002/ece3.1826 (PMC4717349; doi:10.1002/ece3.1826)
Supplement: Supplementary file 4 — Table S3 Eclosion tents dampened fluctuations in soil temperatures within the enclosure. [file ECE3-5-5823-s004.docx]

| Table S3 Eclosion tents dampened fluctuations in soil temperatures within the enclosure. Presented are only instances where significant temperature differences (inside vs. outside tent) were detected (paired t-tests). Significant *P*-values (α = 0.05) are in bold. | | | | | |
| --- | --- | --- | --- | --- | --- |
|  | Temperature (°C) Difference | |  |  |  |
|  | (Inside – Outside) | |  |  |  |
| Area | Mean min | Mean Max | t value | df | *P* |
| 1 | 0.4 (se+0.04) | — | -9.66 | 118 | < **0.0001** |
| 2 | 0.27 (se+0.12) | — | -2.99 | 112 | < **0.0200** |
| 2 | — | -2.18 (se+0.16) | 13.94 | 112 | < **0.0001** |
| 3 | — | -0.56 (se+0.2) | 2.89 | 95 | < **0.0070** |
